# Supplementary material for: High NaCl Concentrations in Water Are Associated with Developmental Abnormalities and Altered Gene Expression in Zebrafish
Source: Int J Mol Sci. 2024 Apr 7;25(7):4104. doi: 10.3390/ijms25074104 (PMC11012806; doi:10.3390/ijms25074104)
Supplement: Supplementary file 1 [file ijms-25-04104-s001.zip › ijms-2837973-supplementary.pdf]

	NaCl	KCl	CaCl2	MgSO4
**E3**	**5 mM**	**0.17 mM**	**0.33 mM**	**0.33 mM**
**5X NaCl**	**25 mM**	0.17 mM	0.33 mM	0.33 mM
**10X NaCl**	**50 mM**	0.17 mM	0.33 mM	0.33 mM
**50X NaCl**	**250 mM**	0.17 mM	0.33 mM	0.33 mM
**100X NaCl**	**500 mM**	0.17 mM	0.33 mM	0.33 mM
**5X KCl**	5 mM	**0.85 mm**	0.33 mM	0.33 mM
**10X KCl**	5 mM	**1.7 mM**	0.33 mM	0.33 mM
**50X KCl**	5 mM	**8.5 mM**	0.33 mM	0.33 mM
**100X KCl**	5 mM	**17 mM**	0.33 mM	0.33 mM
**5X**	5 mM	0.17 mM	**1.65 mM**	0.33 mM
**10X**	5 mM	0.17 mM	**3.3 mM**	0.33 mM
**50X**	5 mM	0.17 mM	**16.5 mM**	0.33 mM
**100X**	5 mM	0.17 mM	**33 mM**	0.33 mM
**5X**	5 mM	0.17 mM	0.33 mM	**1.65 mM**
**10X**	5 mM	0.17 mM	0.33 mM	**3.3 mM**
**50X**	5 mM	0.17 mM	0.33 mM	**16.5 mM**
**100X**	5 mM	0.17 mM	0.33 mM	**33 mM**

Supplemental Table S1. Salt concentration used for treatments.

	Forward primer	Reverse primer	Product size
Shh	CACCTCTCGCCTACAAGCAG	GCTCTTCCCTCGTAGTGGAG	315
Ptc1	TCAGTGAAGCGCATGAACGC	GTCGCTAAGCTTTGAAACCA	130
Actin	TACAATGAGCTCCGTGTTGC	CACCATCACCAGAGTCCAGC	205

Supplemental Table S2. Sequences of primers used in qPCR reactions.
